# Network and Atomistic Simulations Unveil the Structural Determinants of Mutations Linked to Retinal Diseases

**DOI:** 10.1371/journal.pcbi.1003207

**Published:** 2013-08-29

**Authors:** Simona Mariani, Daniele Dell'Orco, Angelo Felline, Francesco Raimondi, Francesca Fanelli

**Affiliations:** 1 Department of Life Sciences, University of Modena and Reggio Emilia, Modena, Italy; 2 Dulbecco Telethon Institute (DTI), Modena, Italy; 3 Department of Life Sciences and Reproduction Sect. of Biological Chemistry and Center for BioMedical Computing, University of Verona, Verona, Italy; Fox Chase Cancer Center, United States of America

## Abstract

A number of incurable retinal diseases causing vision impairments derive from alterations in visual phototransduction. Unraveling the structural determinants of even monogenic retinal diseases would require network-centered approaches combined with atomistic simulations.

The transducin G38D mutant associated with the Nougaret Congenital Night Blindness (NCNB) was thoroughly investigated by both mathematical modeling of visual phototransduction and atomistic simulations on the major targets of the mutational effect.

Mathematical modeling, in line with electrophysiological recordings, indicates reduction of phosphodiesterase 6 (PDE) recognition and activation as the main determinants of the pathological phenotype. Sub-microsecond molecular dynamics (MD) simulations coupled with Functional Mode Analysis improve the resolution of information, showing that such impairment is likely due to disruption of the PDEγ binding cavity in transducin. Protein Structure Network analyses additionally suggest that the observed slight reduction of theRGS9-catalyzed GTPase activity of transducin depends on perturbed communication between RGS9 and GTP binding site. These findings provide insights into the structural fundamentals of abnormal functioning of visual phototransduction caused by a missense mutation in one component of the signaling network. This combination of network-centered modeling with atomistic simulations represents a paradigm for future studies aimed at thoroughly deciphering the structural determinants of genetic retinal diseases. Analogous approaches are suitable to unveil the mechanism of information transfer in any signaling network either in physiological or pathological conditions.

## Author Summary

Incurable retinal diseases causing vision impairments may be due to spontaneous mutations in one component of the visual phototransduction signaling network. Such alterations include the transducin single point mutation G38D associated with the Nougaret Congenital Night Blindness (NCNB). We combined a systems biology approach with atomistic simulations to gain insights into the structural fundamentals of the NCNB disease. Consistent with *in vitro* evidence, mathematical modeling suggests reduced effector recognition and activation as the main determinants of the pathological phenotype. Sub-microsecond molecular dynamics simulations improve the resolution of information, suggesting that such impairment is likely due to disruption of the effector binding cavity. Atomistic simulations also suggest that the observed slight reduction of the RGS9-catalyzed GTPase activity of transducin depends on perturbed inter-protein communication involving the nucleotide. The study highlighted manifold effects of a single point pathogenic mutation, thus paving the way for analogous studies towards a thorough understanding of the structural determinants of genetic retinal diseases.

## Introduction

**Figure 1:**
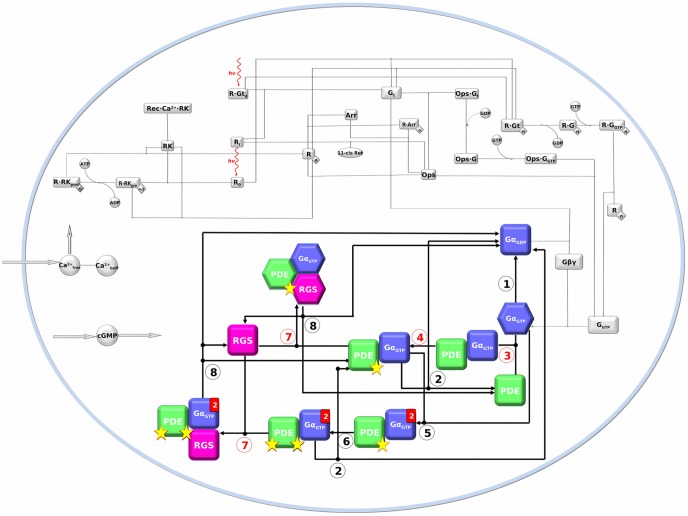
Network structure of the phototransduction model in a rod cell used in the present work. The different forms depict the molecular species involved in the cascade, whereas lines or arrows indicate reversible or irreversible reactions, respectively. Those reactions whose kinetic parameters were changed in this study are numbered and listed accordingly in Table 1. In this respect, red numbers indicate those reactions that were changed to properly model the NCNB pathological conditions. The molecules involved in these reactions, i.e. Gα_GTP_, PDE, and RGS, are blue, green, and magenta, respectively. Filled red rectangles indicate the stoichiometric quantity of the specific molecule in the heteromeric complex, when higher than one. Yellow stars indicate the number of PDE-activated catalytic subunits. Hexagons indicate the molecular species that were used in atomistic simulations.

A number of incurable diseases in the visual system involve one or more components of the phototransduction signaling network (Figure 1). Visual phototransduction is the G protein-mediated process that generates a neuronal signal following light capture by visual pigments in photoreceptor cells (rods and cones). A unique feature of rod cells, the vertebrate photoreceptors dedicated to dim light vision, is the capability to transduce signals from even single photons due to an extremely efficient amplification not paralleled by other signal transduction pathways [1], [2]. The first event in scotopic vision is the absorption of a photon by rhodopsin (R), the cornerstone of family A of the seven-transmembrane G protein coupled receptors (GPCRs), which leads to the formation of the signaling active state (R*) [3], [4]. The latter, in turn, catalyzes the exchange of bound GDP for GTP on the αβγ heterotrimeric G protein transducin (Gt). The GTP-bound α subunit (Gα_GTP_) dissociates from the βγ dimer thus stimulating the activation of phosphodiesterase 6 (PDE), a tetramer made of two nearly identical α and β catalytic subunits and two identical γ subunits [5]. The binding of Gα_GTP_ to the γ subunit of PDE (PDEγ) releases its inhibitory constraint on the catalytic subunits, thus leading to the hydrolysis of guanosine 3′,5′-cyclic monophosphate (cGMP), followed by a rapid closure of the cGMP-gated ionic channels in the outer membrane and a drop in the circulating current. The lowering in intracellular calcium concentration, associated with cell hyperpolarization ultimately signals the presence of light to the secondary neurons of retina. Signaling shutoff includes at least three calcium feedback mechanisms as well as the simultaneous deactivation of Gα_GTP_ and PDE. In this respect, the termination of PDE activation by Gα_GTP_ is achieved when the GTP-bound to Gα is hydrolyzed to GDP by the intrinsic GTPase activity of the protein. The latter process is significantly accelerated by a multiprotein complex containing the ninth member of the Regulators of G protein Signaling (RGS) family, hereafter indicated as RGS [6]. As a result of the GTPase Activating Protein (GAP) action of RGS, the Gα_GDP_ complex re-associates with the βγ dimer restoring the Gα_GDP_-βγ heterotrimer (i.e. Gt).

Misfunctioning of any component of the phototransduction network causes more or less severe vision impairments. Such an example is the Nougaret form of dominant stationary night blindness (Nougaret Congenital Night Blindness, NCNB) caused by a missense mutation, G38D, found in the rod Gα of affected individuals [7]–[9]. Stationary night blindness is not associated with retinal degeneration and is characterized by the inability to see in the dark, whereas daytime vision is largely unaffected [7]–[9]. *In vitro* characterization showed that the aspartate substitution for G38 does not alter the interaction between Gα and Gβγ or activation of transducin by R* [9]. Furthermore, the mutant Gα is characterized by modestly reduced *k_cat_* value for the intrinsic (∼2.5-fold) and RGS-catalyzed (∼5 fold) GTP hydrolyses. In contrast, biochemical data showed that G38D is totally impaired in its ability to bind and activate PDE [9], whereas suction electrode recordings revealed that homozygous Gα_GTP_^G38D−/−^ rods exhibit residual light responses, indicating that the mutation reduced but did not completely abolish effector function [8]. Functional consequences of substituting the homologous amino acid in other G proteins were found to inhibit GTPase activity and to prevent stimulation by GAP in Ras-p21 [10], Gα_i_ [11], Gα_z_ [12], and Gα_s_ [13].

**Figure 2:**
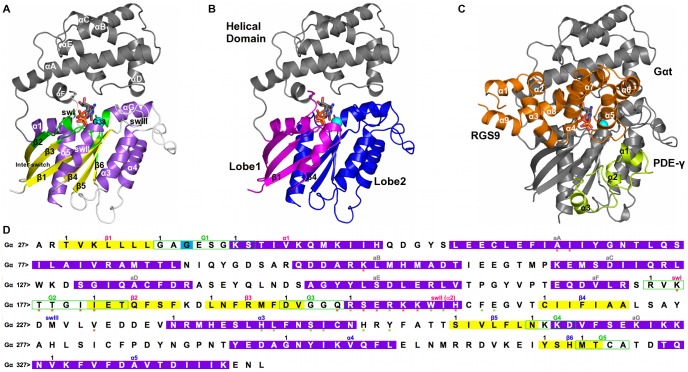
Structure and primary sequence of Gα_GTP_ and Gα_GTP_-RGS-PDEγ^WT^. **A.** The cartoon representation of Gα_GTP_ structure (PDB code: 1TND) is shown. The G protein holds a Ras-like domain and an α-helical domain. The interface between α-helical and Ras-like domain makes the nucleotide binding cleft. The α-helical domain is an orthogonal bundle of six α-helices. The Ras-like domain holds a Rossmann fold, characterized by a 3-layer(αβα) sandwich architecture due to the inversion in the order of the strands β3 and β1 as well as β1 and β4, which are adjacent to each other. The Ras-like domain is colored according to secondary structure (i.e. helices, strands, and loops are, respectively, violet, yellow and white), whereas the α-helical domain is gray. The mutation site is indicated by a cyan sphere centered on the Cα-atom. The GTP nucleotide is represented by sticks colored by atoms type. The nucleotide docks into a binding site contributed by the β1/α1, α1/β2 (αF/β2 in the Gα proteins), β3/α2, β5/α4 and β6/α5 loops. These are ultraconserved regions also called G boxes 1–5 (G1–G5, colored green). G2 is also called swI (α1/β2 loop (αF/β2 loop in the Gα proteins)), whereas G3 is part of the switch II (swII, or β3/α2 loop, plus the α2-helix). The β2/β3 hairpin in between swI and swII is also called inter-switch. The β4/α3 loop, which is not a G box, is also called swIII [19]. In Gα proteins, the two domains are connected by two loops, linker 1 or α1/αA loop and linker 2 or αF/β2 loop; the latter corresponds to swI. **B.** According to computational experiments [20], the strands β1 and β4 divide the conserved domain into two dynamically distinct lobes, lobe 1 (i.e. the N-terminal half of the domain, colored magenta) and lobe 2 (i.e. the C-terminal half of the domain, colored blue). **C.** The cartoon representation of the complex involving Gα_GTP_ (gray), RGS (i.e. the RGS domain of RGS9, amino acids 286 to 418, orange), and PDEγ (green) is shown (PDB code: 1FQJ [16]). In deep detail, the RGS domain is a bundle of nine α-helices, configured into two subdomains: an N- and a C-terminal region holding an orthogonal bundle architecture (helices α1, α2, α3, α8 and α9), and a prototypical right-handed, antiparallel four-helix bundle (helices α4, α5, α6 and α7). PDEγ (residues 46–87) comprises three short α-helices and an N-terminal loop region that originates near the C-terminus and winds over helices α1 and α2. **D.** The primary sequence of Gα is shown. Helices, strands, and loops are, respectively, violet, yellow, and white. The G boxes are delimited by green boxes. Black numbers on the left side of the alignment refer to the sequential numbering, whereas black numbers above the sequences indicate the beginning of a secondary structure/G-box motif. An arbitrary numbering of each residue was set, characterized by the label of the secondary structure segment followed by the amino acid position within the segment. In those cases where the G-boxes overlap with the secondary structure segment, positions refer to the G-boxes. Orange and green stars mark, respectively, RGS and PDEγ recognition sites.

Thus, a single-point mutation in Gα seems to elicit a multitude of effects not entirely clarified by *in vitro* and *in vivo* experiments and likely involving more than one component in the phototransduction signaling network. In this framework, to gain insights into the molecular bases of the NCNB disease, we integrated the information from *in vitro*/*in vivo* experiments with systems-based and atomistic modeling. The systems-based approach relied on a comprehensive quantitative model of phototransduction in rod cells that explicitly includes most of the molecular components of the cascade [14] (Figure 1). In this study, that model was extended to the NCNB pathological phenotype, thus highlighting those reactions and intermolecular interactions perturbed by the Gα mutation. The molecular systems involved in those reactions were subjected to atomistic Molecular Dynamics (MD) simulations and included: wild type and mutated Gα_GTP_ taken both in their isolated forms (i.e. Gα_GTP_^WT^ and Gα_GTP_^G38D^) and in the ternary complex with RGS and PDEγ (Gα_GTP_-RGS-PDEγ^WT^ and Gα_GTP_-RGS-PDEγ^G38D^) (Figure 2).

## Results

### Mathematical modeling of the NCNB phenotype

**Figure 3:**
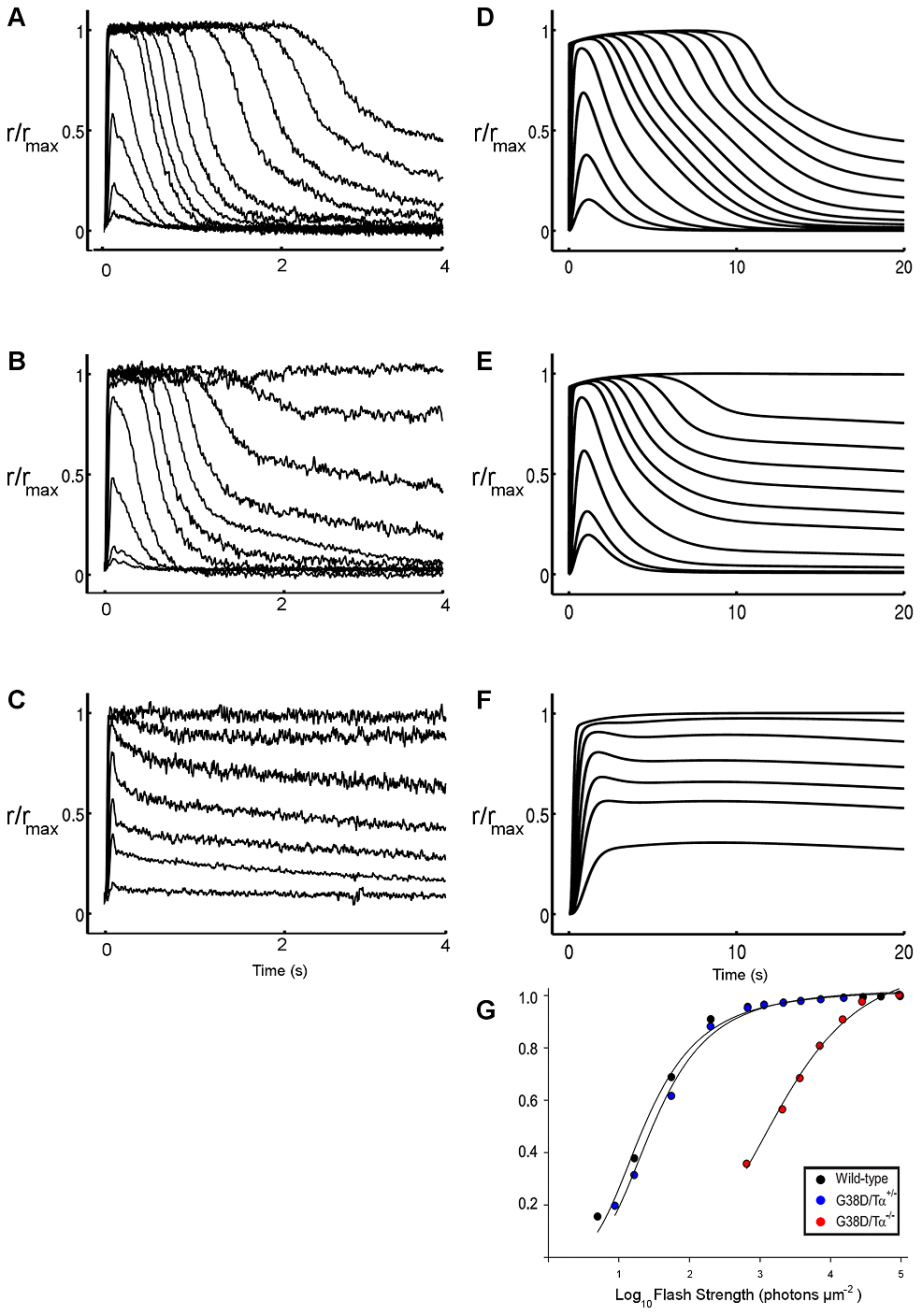
Flash responses from wild type, Gα_GTP_^G38D+/−^, and Gα_GTP_^G38D−/−^ rods. Experimental (A, B, C) *versus* simulated (D, E, F) responses to flashes of increasing intensities from wild type Gα_GTP_^WT+/+^ (A, D), heterozygous Gα_GTP_^G38D+/−^ (B, E) and homozygous Gα_GTP_^G38D−/−^ (C, F) rods are shown. Experimental data, i.e. published in Moussaif et al. [8] and provided by Marie E. Burns, refer to mice rods exposed to flashes ranging from 5 to 97000 photons µm^−2^ (A and B) or from 650 to 94000 photons µm^−2^ (C). Simulated responses derive from the model of an amphibian rod stimulated with the same light intensities as *in vitro* recordings. The pathological Gα_GTP_^G38D+/−^ and Gα_GTP_^G38D−/−^ models were generated by changes in the kinetic parameters *kP1*, *kP2* and *kRGS1* as described in the text. The dissimilar species justify the time scale difference between *in vitro* and *in silico* experiments. The responses were normalized with respect to the maximum photocurrent. **G.** Normalized simulated light response amplitude is plotted as a function of flash strength. For comparison to *in vitro* data, see Figure 5B in Moussaif et al. [8]. Flash intensities are the same as in D, E and F.

We presented a dynamical model of the phototransduction signaling network made up of ordinary differential equations, which describe the reactions and their kinetic parameters [14]. The working model used in this study includes also the dynamic scaffolding reactions between dark rhodopsin and Gt [15]. Herein, such model was further extended to describe the heterozygous (Gα_GTP_^G38D+/−^) and homozygous (Gα_GTP_^G38D−/−^) mutated conditions of Gα_GTP_^G38D^. This was accomplished by introducing the mutated G protein as an explicit new molecule and adding all the relative reactions in the phototransduction cascade, which concerned the Gα_GTP_^WT+/+^ status (Table 1, see Methods). The output of mathematical simulations (i.e. change in photocurrent with respect to dark value, ΔJ) was analyzed and compared with the photoresponses of rods from wild type and transgenic mice (Figure 3A, 3B, and 3C). It is worth noting that, due to the significant difference in the species between *in vitro* (i.e. mammals, Figure 3A, 3B, and 3C) and *in silico* (i.e. amphibian rods [14], Figure 3D, 3E, and 3F) experiments, the time scales of the photoresponses is different, thus allowing for semi-quantitative comparisons.

**Table 1:** Reactions that were changed in the background of the G38D mutation.

N^a^	Reaction Equation	Description^b^	O.P.^c^	Min^d^	Max^d^	Effect^e^
**1**	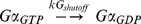	Intrinsic Gα_GTP_ GTPase activity	0.05	×2	0	None
**2**	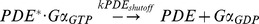	Intrinsic Gα_GTP_-PDE GTPase activity	0.033	×2	0	Slowed recovery when = 0
**2**	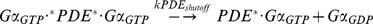	Intrinsic Gα_GTP_-PDE- Gα_GTP_ GTPase activity	0.033	×2	0	Slowed recovery when = 0
**3**	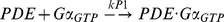	Binding of Gα_GTP_ to inactive PDE	5.5e-2	×2	×10^9^	Slowed activation, lower sensitivity and recovery with changes >×10^6^
**4**	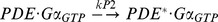	Activation of one PDE catalytic subunit	940.7	×2	×10^4^	Slowed activation phase with changes >×10^3^
**5**	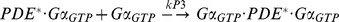	Binding of Gα_GTP_ to Gα_GTP_-PDE	1.498e-9	×2	×10^5^	None
**6**	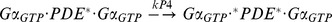	Activation of the PDE tetramer	21.09	×2	×10^5^	None
**7**	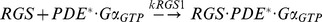	Binding of RGS9 to Gα_GTP_-PDE	1.57e-7	×2	×15	Slowed recovery phase
**7**	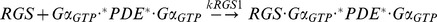	Binding of RGS9 to Gα_GTP_-PDE-Gα_GTP_	1.57e-7	×2	×15	Slowed recovery phase
**8**	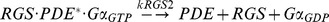	GAP activity and disruption of the complexes	256.07	×2	×10^5^	Increase in saturating phase for bright flashes with changes >10^2^
**8**	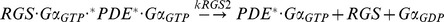	GAP activity and disruption of the complexes	256.07	×2	×10^5^	Increase in saturating phase for bright flashes with changes >10^2^

a Reaction number, corresponding to that in Figure 1.

b Type of reaction.

c Original value of the parameter governing the reaction kinetics in the mathematical model (s^−1^) [14].

d Minimum and maximum reductions the considered parameters were subjected to in the present study, in order to model the mutated conditions; when “Max R” was 0, the parameter was ultimately set to zero.

e Effect of these reductions.

The results obtained with our Gα_GTP_^WT+/+^ model were in remarkable agreement with *in vitro* recordings on wild type cells (Figure 3A and 3D).

In order to fit the models onto the pathological NCNB condition, the rates of a number of reactions involving Gα_GTP_^G38D^ were systematically reduced by tuning the relative kinetic parameters in decreasing steps (Table 1, Figure 1, see Methods). The reductions were combined into specific heterozygous and homozygous models. At the end, the best fit with electrophysiological recordings of the mutant cells was obtained by making changes in the following reactions:

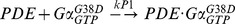

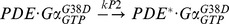

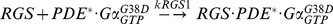

describing, respectively, a) the binding of one molecule of Gα_GTP_ to one inactive PDE subunit, b) activation of the Gα_GTP_-PDE complex, and c) binding of the RGS complex to a PDE tetramer with one active subunit. The relative changes in the parameters were 35000-fold reduction in *kP1* and *kP2* and 2-fold reduction in *kRGS1*, with respect to the wild type value. Following such changes, heterozygous cells show a similar behavior to wild type cells under dim flash responses but elongated recovery under brighter flashes associated with slight loss in sensitivity (i.e. a 40% brighter flash is required to generate a half-maximal response, I_o_; Figure 3G and Figure 5B in Moussaif et al. [8] and Table 2). In contrast, homozygous cells show marked decrease in sensitivity to light (50-fold brighter I_o_, Table 2) and impaired response recoveries for all flash intensities.

**Figure 5:**
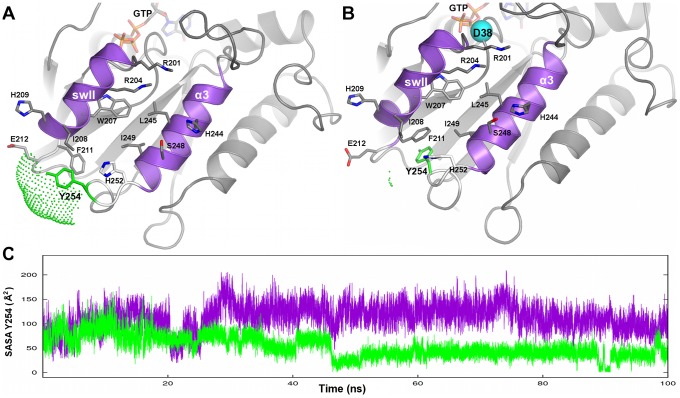
SASA time series. The SASA computed on Y254 (in the α3/β5 loop) is shown. Cartoons of the Gα_GTP_^WT^ (A) and Gα_GTP_^G38D^ (B) snapshots halfway through the simulation (frame 50000^th^) are shown, which are zoomed on the swII/α3 cleft, the primary recognition site for PDEγ. The Gα residues directly involved in PDEγ recognition (i.e. marked by green stars in the primary sequence in Figure 2) are shown in sticks. The atom color code is grey for carbon, blue for nitrogen, and red for oxygen. The residue Y254, on which the SASA index was computed, is green. The cyan ball in B indicates the mutation site. C. The time series of the SASA index calculated on Y254 along the 100 ns trajectories of Gα_GTP_^WT^ (violet) and Gα_GTP_^G38D^ (green) are shown.

**Table 2:** *in vitro* and *in silico* flash responses.

	I_0_(photons/µm^2^)^a^		Time to Peak^b^	
	*in vitro*	*in silico*	*in vitro*	*in silico*
**Gα_GTP_^WT^**	48.3±2.1	23.64	111±6	0.96
**Gα_GTP_^G38D+/−^**	77.4±6.0 (1.6)	31.5 (1.3)	109±9 (1)	0.975 (1.01)
**Gα_GTP_^G38D−/−^**	2296±125 (47.5)	1854 (78.4)	192±43 (1.7)	2.345 (2.4)

a Flash strength that elicited a half-maximal response. The time scales for *in vitro* [8] and *in silico* measurements are ms and s, respectively.

b Time to Peak of the flash.

*In vitro* data were taken from the literature [8], while *in silico* data are the outcome of mathematical simulations done in this study. In brackets, the ratios to the wild type value are shown.

Same strengths of flashes delivered to the cells in simulations and electrophysiological recordings [8] result in quantitative differences concerning time scales and sensitivity of the photoresponses, likely due to the different species considered. The higher ΔJ elicited by the dimmest flash in computational experiments compared to electrophysiological recordings is exemplar in this respect (Figure 3A and 3D). Differences between the cellular properties of mammals and amphibian rods include temperature and volume, which likely influence initial conditions and concentrations of the molecular species involved in signal transduction [5].

It is worth noting that, in the actual mathematical model, up to two Gα_GTP_ can bind, and in turn activate, either one of the catalytic subunits of PDE, hence leading to a 2∶1 stoichiometry. Nevertheless, as previously discussed [14] (see also Table S4 therein), the 2∶1 Gα_GTP_∶PDE complex is detectable only in the presence of light flashes with intensities in the order of 10^5^ photons/µm^2^ and, even then, their presence is negligible. As a confirmation of those results, deep reductions of *kP3* and *kP4* (regulating, respectively, the binding of the second molecule of Gα_GTP_ to Gα_GTP_-PDE and the activation of both catalytic subunits of the PDE tetramer), in the background of any of the test models used in this study, did not elicit any change in the photoresponse (Table 1). We cannot, however, exclude that this was due to an inaccurate modeling of this part of the network, whose biochemical detail remains mainly unknown. For example, a finer treatment of the allosteric and regulatory mechanisms may be necessary to recover the role of the second PDE subunit [14]. This might also explain why, with the changes in *kP1*, *kP2*, and *kRGS1* necessary to reproduce Gα_GTP_^G38D−/−^ photoresponses, loss in sensitivity and delay of the time to peak are more marked in simulated responses compared to *in vitro* ones (78- vs 47- and 2.4- vs 1.7-fold, respectively, see Table 2).

In line with the statements above, we relied on the fact that, in the present model, changes in *kRGS1* and *kRGS2* only affect the formation and activity of the 1∶1∶1 Gα_GTP_-RGS-PDE complex. Noteworthy, as shown in Table 1, *kRGS2* is a rather coarse parameter, as it describes the RGS-catalyzed GTPase activity in both Gα_GTP_-RGS-PDE and Gα_GTP_-RGS-PDE-Gα_GTP_ complexes as well as disruption of these complexes. For this reason, we couldn't use the mathematical model to properly evaluate mutational effects on the GAP activity of RGS in the Gα_GTP_-RGS-PDE complex.

In summary, consistent with electrophysiological recordings [8] but not with earlier biochemical data [9] mathematical modeling highlights reduction of both PDE binding and activation as the major mutation-induced perturbations in the visual phototransduction signaling network. A marginal reduction in RGS binding helped as well in reproducing the electrophysiological phenotype. On these bases, insights into the structural determinants of such perturbations were searched by atomistic MD simulations targeting both wild type and mutated Gα in its isolated form and in ternary complex with both RGS and PDEγ.

### Atomistic simulations on the Gα_GTP_ system: The G38D mutant affects the swII/α3 cleft, primary determinant in PDEγ binding

Atomistic simulations were firstly carried out on the isolated Gα_GTP_ in its wild type and mutated forms (Gα_GTP_^WT^ and Gα_GTP_^G38D^, respectively).

The mutation site is the third position of the G box 1 (i.e. G1:3, see Figure 2 and its legend for description and visualization of the G protein regions as well as for explanation of the position-based numbering). Incidentally, the G boxes are five ultra-conserved regions of the Ras-like domain involved in nucleotide binding (Figure 2). Such mutated position is not involved in backbone-mediated H-bonding interactions with the nucleotide neither in the wild type nor in the mutant, (Supplementary Figure 1A (Figure S1A)). The interaction pattern of the nucleotide remains almost unchanged in the two forms following MD simulations, as also indicated by the patterns of interaction energies between GTP and surrounding residues (Figure S2). Collectively, these data are consistent with the results of *in vitro* evidence that the mutation elicits a marginal effect on the intrinsic GTPase activity of the protein [8], [9].

**Figure 4:**
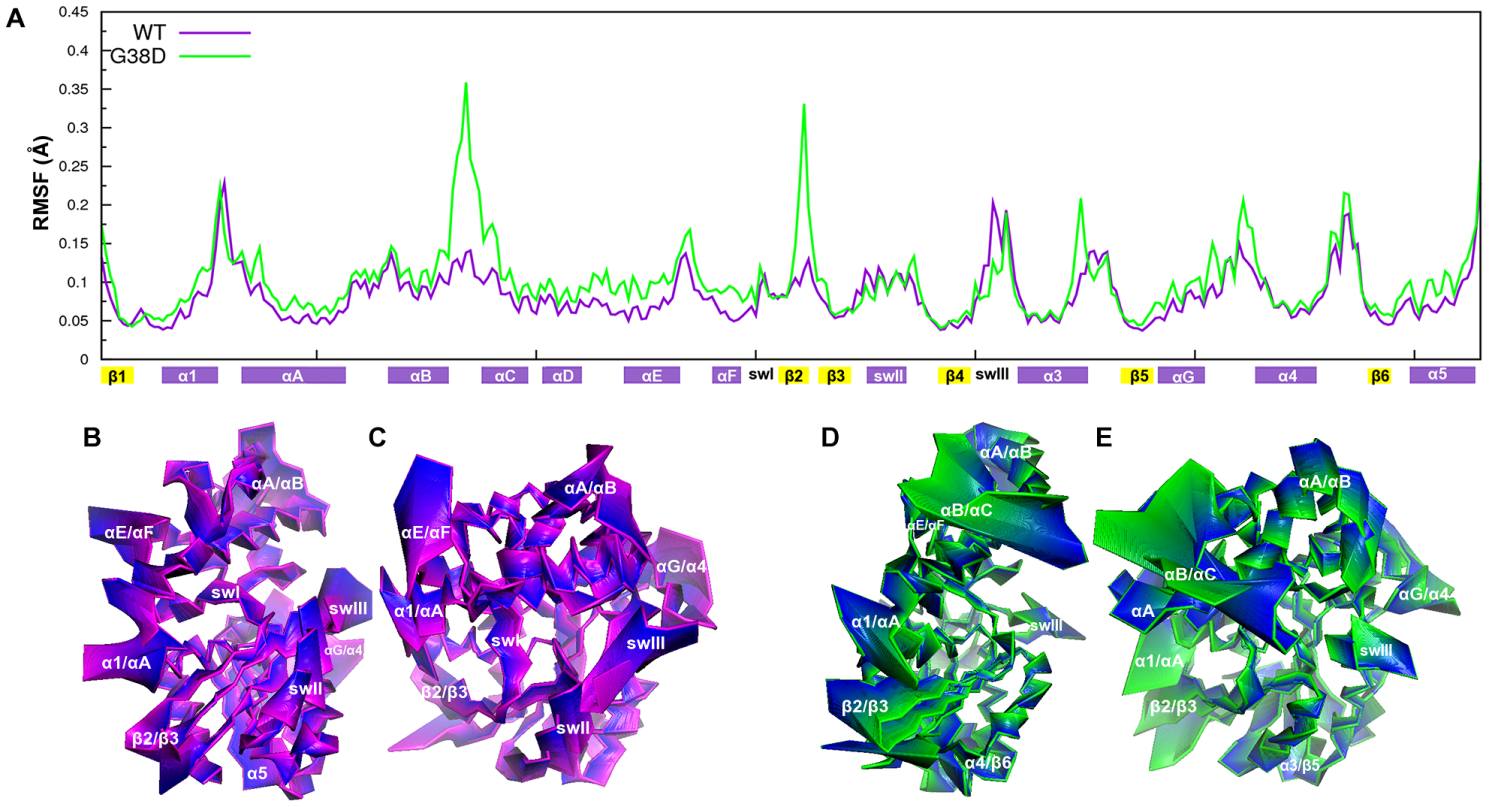
RMSF profiles and PCA projections. **A.** The Cα-RMSF profiles from MD trajectories of Gα_GTP_^WT^ (violet) and Gα_GTP_^G38D^ (green) are shown. They refer to the 100000 frames constituting the 100 ns trajectory. The secondary structure elements are shown on the abscissa, following the Noel's nomenclature [19]. **B, C, D, E.** The Cα-atom projections along the linear combination of the ED analysis-derived principal components, which describe the essential subspace of the trajectories of Gα_GTP_^WT^ (B and C) and Gα_GTP_^G38D^ (D and E) are shown (see text for an explanation of ED). The number of PCs used was 111 for Gα_GTP_^WT^ and 74 for Gα_GTP_^G38D^. Cα-atom displacements are highlighted by color ranges from violet to blue for Gα_GTP_^WT^, and from green to blue for Gα_GTP_^G38D^.

In contrast to lack of local structural effects, the G38D mutant turned out to affect the intrinsic dynamics of the protein. Indeed, the Cα-atom Root Mean Square Deviation (RMSD) of the mutant is higher than that of the wild type especially over the second half of MD simulation (Figure S3). As expected, Cα-atom fluctuations evaluated in terms of Root Mean Square Fluctuations (RMSFs) show peaks of flexibility in the loops connecting the elements of secondary structure, especially those in the α-helical domain. This effect is greater in the mutant than the wild type (Figure 4). In line with RMSFs, in the mutant form, selected portions of the protein show significant enhancements in their collective motions as inferred from the Principal Component Analysis (PCA) of the trajectories. These portions include linker1, αB/αC loop, αC, αE, αF, inter-switch, and C-term of α3 (Figure 4).

To investigate whether mutation-induced changes in intrinsic dynamics may affect Gα portions deputed to RGS and/or PDEγ recognition, we monitored the solvent accessibility of all the RGS and PDEγ recognition sites on Gα (indicated, respectively, by orange and green stars in Figure 2), finding more marked effects on the PDEγ sites, in particular Y254 (in the α3/β5 loop). The latter is, indeed, exposed to the solvent in the wild type but buried in the mutant form where it is permanently involved in inter-helical interaction with F211^(s2:11)^, another PDEγ recognition site that becomes no longer available to PDEγ as well (Figure 5). In line with these observations, Functional Mode Analysis (FMA, see Methods) found that the Solvent Accessible Surface Area (SASA) of Y254 is correlated with the modes describing the essential subspace of Gα. Incidentally, the essential subspace (ES) is given by a variable number of eigenvectors whose associated eigenvalues account for 90% of the total variance of the Cα-atom displacements in a trajectory. The correlation is already present in the wild type but increases in the mutant form (i.e. the correlation coefficients are 0.73 and 0.86, respectively). Differences in functional modes between wild type and mutant amplify when considering only the first principal component (PC1); in fact, the correlation remains still significant for the mutant (i.e. 0.74) but it drops for the wild type (i.e. 0.39). Collectively, FMA is suggestive of a functional link between protein dynamics and structural environment of Y254. Thus, mutation-induced burying of Y254 results in deformation of the swII/α3 cleft, which is the primary determinant in PDEγ binding.

We also investigated mutational effects on the structural communication features of Gt by the Protein Structure Network (PSN) analysis, a product of graph theory applied to protein structures (see Methods). The analysis searched for mutation-induced changes in network components (e.g. nodes, hubs, links, shortest communication pathways, etc) on the MD trajectories. The comparative analyses of the Protein Structure Graphs (PSGs) of wild type and mutated Gα revealed a slight reduction in number of nodes, hubs, and links in the G38D mutant compared to the wild type (Table 3). In contrast, the number of communication pathways and their average length increases in the mutant compared to the wild type. In line with this trend, the maximal, minimal, and average strengths reached by the totality of links in the paths tend to be higher in the mutant compared to the wild type.

**Table 3:** Network parameters.

	Gα_GTP_^WT^	Gα_GTP_^G38D^	Gα_GTP_-RGS-PDE^WT^	Gα_GTP_-RGS-PDE^G38D^
Imin^a^	3.24	3.23	3.52	3.58
Hubstot^b^	34	33	51	51
Nodes 1^st^ Cls^c^	228	192	291	285
Hubs 1^st^ Cls^c^	34	28	47	46
Links 1^st^ Cls^c^	304	265	396	394
Number of Paths^d^	268	330	1930	975
1^st^ Cluster Pop^d^	57	72	1819	924
2^nd^ Cluster Pop^d^	27	37	80	36
3^rd^ Cluster Pop^d^	26	34	22	7
4^th^ Cluster Pop^d^	22	23	22	4
5^th^ Cluster Pop^d^	13	19	22	/
Max Length^e^	7	9	16	11
Avg Length^e^	5.4	5.6	7.7	6.1
Max Freq^f^	83.6	82.7	85.2	82.3
LengthMaxFreq^f^	5	5	5	5
Max Score^g^	1.0	1.0	1.0	1.0
Min Score^g^	0.4	0.6	0.4	0.5
Avg Score^g^	0.9	0.9	0.4	1.0
Max SumWgt^h^	79.1	89.2	155.3	127.8
Min SumWgt^h^	26.2	36.1	32.5	32.7
Avg SumWgt^h^	51.1	54.9	77.1	62.9
Max AvgWgt^i^	11.3	12.1	12.3	12.5
Min AvgWgt^i^	4.3	5.9	5.4	5.5
Avg AvgWgt^i^	7.9	8.3	8.8	8.9

a Imin values (%) employed for the four simulated systems.

b Total number of hubs.

c Number of nodes, hubs and links in the most populated node cluster.

d Total number of paths characterized by frequency ≥30% and number of paths in the first five most populated path clusters.

e Maximum and average path length excluding the two extremities.

f Maximum path frequency and length of the maximum frequency path (i.e. excluding the extremities).

g Maximum, minimum and average correlation score.

h Maximum, minimum and average path strength obtained by summing the interaction strengths of the links constituting the path.

i Maximum, minimum and average path strength obtained by summing the interaction strengths of the links constituting the path and dividing such sum by the number of links involved in the path.

**Figure 6:**
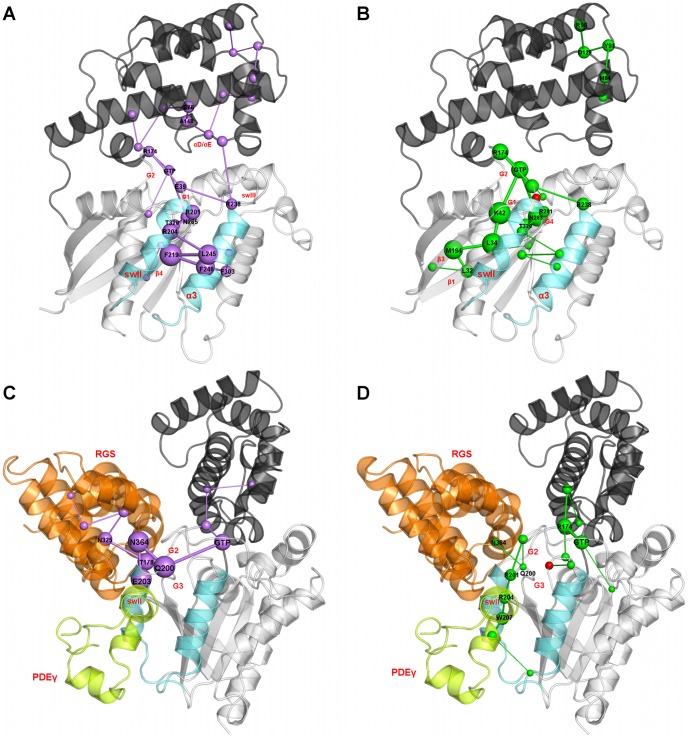
Global and coarse view of the communication pathways. The global meta paths regarding Gα_GTP_^WT^ and Gα_GTP_^G38D^ in their free state (A and B panels, respectively) as well as in ternary complex with both RGS and PDEγ (C and D panels, respectively) are represented, colored violet and green, respectively. The width of each link is proportional to *r*, while the sphere diameter is proportional to the average *r* of the connecting link (see Methods for *r* definition). The α-helical and Ras-like domains are dark and light gray, respectively, the PDEγ binding site on Gα is aquamarine, RGS is orange and PDEγ is lemon-green. The mutation site is indicated by the red sphere.

To infer a global and coarse view of mutation-induced changes in the communication pathways of Gα we drew global meta paths, i.e. assemblies of the most recurrent nodes and links in the pool of paths characterized by frequency ≥30% (Figure 6A and 6B, see Methods). In this respect, whereas the wild type is characterized by nucleotide-mediated paths at the interface between Ras-like and α-helical domain, in the mutant form, inter-domain pathways are less frequent as also highlighted by the distribution of linked-node fragments (Figures 6 and S4). This inter-domain uncoupling may be in part related to the fact that, in the mutant, selected portions of the α-helical domain undergo increases in essential dynamics compared to the wild type (Figure 4). Noteworthy, this trend is also evident in the meta paths computed on the sub set of paths made by ≥50% of conserved amino acids (Figure S5A and S5B). Differently from the wild type, in the mutant the most frequent nucleotide-involving pathways transverse essentially β1 and β3 rather than the swII/α3 interface, which is the primary PDEγ binding cleft (Figure 6A and 6B).

In summary, in spite of the lack of significant differences in the interaction pattern of GTP between wild type and mutated forms, Gα_GTP_^G38D^ is characterized by increased flexibility of the α-helical domain compared to wild type, which reflects on an apparent inter-domain uncoupling in terms of shortest communication pathways. In contrast, in the wild type, inter-domain pathways localized on the nucleotide binding site cover a significant part of the structural communication modes. Above all, the most significant effect of aspartate substitution for G38 is a structural perturbation in the swII/α3 cleft participating in the PDEγ binding site. A marker of such perturbations is the solvent accessibility of Y254, which in the mutant becomes buried and no more available for PDEγ interaction, shielding also F211^(s2:11)^ from effector binding.

### Atomistic simulations on the Gα_GTP_-RGS-PDEγ ternary complex: The G38D mutant affects the communication between nucleotide binding site and RGS

Atomistic MD simulations on monomeric Gα_GTP_ highlighted long-distance mutational effects on the Gα regions deputed to PDEγ recognition likely related to the reduced PDE binding and consequently activation inferred from both mathematical simulations and *in vitro* experiments [8].

In order to clarify ambiguities by *in vitro* experiments on the GAP activity of RGS towards the G38D mutant [8], [9], which couldn't be properly addressed by mathematical simulations, wild type and mutated Gα were simulated also in the context of the ternary complex with PDEγ and RGS. In this respect, MD simulations on the Gα_GTP_-RGS-PDEγ^G38D^ ternary complex are justified by the fact that the mutated Gα holds a residual binding to PDEγ [8].

Comparing the dynamics and structural communication features of all the components of the ternary complex in the presence of either wild type or mutated Gα served to infer the effects of the G38D mutation on different structural aspects such as: a) communication and interaction features of the nucleotide, b) intrinsic dynamics of each component of the complex, and c) inter-protein communication.

In line with simulations on monomeric Gα_GTP_, simulations on the ternary complex show that the interaction pattern of the nucleotide is substantially similar in wild type and mutated Gα (Figure S1 and S2), thus not providing any clue on mutational effects on the GAP activity of RGS.

As for the intrinsic dynamics of the three proteins in the complex, differently from PDEγ, Gα_GTP_ and RGS are characterized by low mobility in terms of RMSDs, (Figure S3). The higher mobility of PDEγ is likely due to the poor intramolecular and intermolecular tertiary contacts made by such protein, which is a 42-amino acid fragment of a small subunit. In deep detail, as for Gα_GTP_, the wild type and mutated forms do not differ significantly in terms of RMSDs or RMSFs; major differences concern only the swIII region, which fluctuates less in the mutant than in the wild type (Figures S3 and S6). In line with such behavior, the overlap between the ES of the two Gα forms in the context of the heterotrimer is quite high (0.90), the essential motions of β2/β3 loop and swIII contributing to such differences (Figure S6). RGS shows low mobility as well, its intrinsic flexibility being comparable in the complexes with wild type and mutated Gα (Figure S3). In contrast, the intrinsic flexibility of PDEγ is higher in the complex with mutated Gα_GTP_ than in the complex with wild type Gα_GTP_ (Figures S3 and S7). This suggests that the pathogenic Gα mutation increases the intrinsic flexibility of PDEγ, which would imply increased instability of the PDEγ-Gα_GTP_ interface.

According to the crystal structure of the heterotrimeric complex, RGS does not contribute directly to the active site by donating residues or through water-mediated interactions [16]. It is rather thought that RGS would increase the GTP hydrolysis rate by stabilization of the Gα switch regions in their transition state conformation and orientation of the critical Gα carbonyls used to position the nucleophilic water [16], [17]. Thus, RGS action is likely due to inter-protein structural communication. On these bases, possible structural relations with the postulated mutation-induced reduction of the GAP activity of RGS were searched by the PSN analysis. Significant differences between the two simulated ternary complexes could be inferred from the analysis of the shortest communication pathways, which were almost halved in the mutated complex compared to the wild type (Table 3). Remarkably, more than 60% of the communication paths that characterize the wild type form hold the GTP-Q200^(G3:5)^-R:N364 fragment of linked nodes, which is completely absent in the mutant (Figure S4). The global meta path representation clearly shows that the most significant communication in the Gα_GTP_-RGS-PDEγ^WT^ involves GTP, Q200^(G3:5)^, R:N364, and E203^(s2:3)^ (i.e. the GTP-Q200^(G3:5)^-R:N364-E203^(G3:8)^ meta fragment of linked nodes; Figure 6C and 6D). Remarkably, the GTP-Q200^(G3:5)^-R:N364 connection found in the wild type form is essential for the GAP activity of RGS [16]. Such connection is no longer present in the mutant. In line with path fragment distribution, the most representative nucleotide-mediated paths in the Gα_GTP_-RGS-PDEγ^G38D^ complex are intra-Gα located (Figure 6C, 6D, and S4). These differences between wild type and mutant forms are strengthened by the meta paths computed on the sub set of paths made by ≥50% of conserved amino acids (Figure S5). Thus, nucleotide-mediated paths involving the RGS-Gα interface are few and characterized by the D38^(G1:3)^-Q200^(G3:5)^-R:N364 fragment of linked nodes (Figure S4). Another difference concerning the structural communication of wild type and mutated Gt is that, whereas for the wild type some (4%) of the shortest pathways describe a communication between Gα and PDEγ, in the mutant form such communication could not be found, likely due to the increased flexibility of the effector subunit.

In summary, atomistic simulations on the ternary complexes highlight a possible disturbing effect of the pathogenic mutation on the GAP activity of RGS. This would act, at least in part, by destabilizing the Q200^(G3:5)^-mediated communication between GTP and R:N364. Finally, they strengthen the influence of the mutation on the G protein-effector interface, in line with electrophysiological recordings and mathematical simulations.

## Discussion

Mutations in any components of the visual phototransduction signaling network may cause more or less severe impairments in vision. Because of the complexity of such network, any alteration of one of the cascade components would lead to unpredictable and not easily determinable results. Thus, a monogenic disease such as NCNB, considered in this study, can result from perturbations not circumscribed to the mutated protein but involving also other members of the network. In this framework, modeling the effects of mutation by systems-based approaches serves to infer how the pathogenic signal propagates through the network and which molecular species are involved. When possible, the latter information is passed to atomistic simulations to gain insights into the structural determinants of the disease.

In this study, we combined visual phototransduction modeling with atomistic simulations to thoroughly investigate the defect associated with the NCNB-causing G38D mutation of Gα. The mathematical model of visual phototransduction was able to reproduce the key features of the behaviors of heterozygous and homozygous rods typical of the NCNB disease. This could be possible upon reducing the constants governing: a) the binding of Gα_GTP_ to PDE, b) the activation of the catalytic activity of PDE, and c) the binding of the RGS complex to a PDE tetramer with one active subunit. In fact, a strong reduction in PDE recognition and activation by Gα_GTP_ coupled to a two-fold reduction in the RGS binding constant was essential to reproduce the visual responses in Nougaret patients, while decreasing the intrinsic or RGS-catalyzed GTPase activities did not seem to have a significant effect. Thus, mathematical modeling emphasized the formation and activation of the Gα_GTP_-PDE complex as the processes more significantly affected by the aspartate substitution for G38 in Gα, in line with electrophysiological recordings [8]. MD simulations on monomeric Gα_GTP_ suggest that such reduction in the PDEγ binding ability of mutated Gα is likely due to altered dynamics of the protein associated with changes in the architecture of the swII/α3 cavity, essential recognition point for PDEγ. A detrimental effect of the mutation on such cavity had been also postulated based upon crystallographic analyses [16]. We ended up independently with this conclusion by individuating also the main actors of this structural effect. In this framework, the Y254 position seems to be particularly sensible to the concerted motions of the protein triggered by the mutation. Indeed, deformation of the swII/α3 cavity results in the burying of Y254 in the α3/β5 loop, preventing both the Y254 itself and F211^(s2:11)^ from being available to PDEγ. We also speculated that mutation-induced reductions of the catalytic activity of PDE may derive from the formation of an improperly assembled Gα_GTP_-PDEγ complex. Along this line, also in the ternary complex with RGS and PDEγ the mutation exerts a long distance effect on the effector binding site resulting in increase in the intrinsic flexibility of PDEγ and lack of communication pathways at the Gα-PDEγ interface. Another clear effect of the Gα mutation is the incapacity to form a stable Q200^(G3:5)^-mediated communication between the nucleotide and N364 of RGS. Such communication is instead present in the wild type and is necessary for the GAP action of RGS [16].

In conclusion, the main structural effects of the G38D mutation turned out to be deformation of the primary effector binding site in monomeric Gα and enhanced flexibility of PDEγ in the ternary complex, which would destabilize the Gα-effector interface. Collectively these effects are connectable with the impaired effector recognition and activation shown by *in vitro* experiments. Finally, the pathogenic mutation of Gα seems to affect the communication between RGS and nucleotide essential for the GAP activity, thus suggesting that the observed slight reduction of the RGS-catalyzed GTPase activity is a matter of perturbed inter-protein communication.

This study extends the dynamic model of visual phototransduction to the pathological NCNB phenotype and provides insights at the atomic level into the structural bases of the disease. This is an example of a thorough computational investigation employing different scales of description, an approach which should be pursued to unveil the structural determinants of genetic retinal diseases. Analogous approaches are suitable to infer the mechanisms of information transfer in any signaling network either in physiological or pathological conditions.

## Methods

### Mathematical modeling

The mathematical model of rod phototransduction (BioModels ID: BIOMD0000000326), employed in the present work for numerical simulations of the phototransduction cascade includes 91 reactions, 71 molecular species, and 63 parameters and was previously developed and validated over a wide range of experimental data on normal and genetically modified rods [14]. The rate of change of the molecular species are monitored by calculating, at given time steps, their rates of production and consumption [14]. The original model was recently modified to account for the postulated R-Gt precoupling in the dark [15], leading to the inclusion of the following reactions, which describe: a) the dynamic formation and b) dissociation of dark R-Gt complexes:

*a*)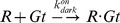 and its reverse
*b*)
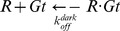

The parameters were calculated as relative kinetic constants, following the relationships 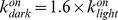 and 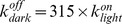 taken from Surface Plasmon Resonance (SPR) experiments [15]. This led to a ≈20% of Gt to be dynamically precoupled to Rin the dark, consistent with published data [15].

In this study, we built a dynamic model able to describe the Gα_GTP_^G38D+/−^ and Gα_GTP_^G38D−/−^ mutated conditions of Gα_GTP_^G38D^ by introducing the mutated G protein as an explicit new molecule and adding all the relative reactions in the phototransduction cascade, which concerned the Gα_GTP_^WT+/+^ status (Table 1). The concentration of the new species (Gα_GTP_^G38D^) was obtained by using its ratio to the normal concentration of Gα in wild type cells taken from expression levels in transgenic mice for the mutation [8]. Therefore, the total level of Gα (25% of which is Gα_GTP_^G38D^) in heterozygous cells is the same as in wild type rods, while only 35% of the native levels are found in homozygous cells for the mutation. The levels of all the other proteins were kept unchanged and the mutation was assumed to have no effect on rhodopsin-Gα_GTP_^G38D^ binding [8], [9].

In order to fit the models onto the pathological NCNB condition, the rates of a number of reactions involving Gα_GTP_^G38D^ were systematically reduced by tuning the relative kinetic parameters in decreasing steps (Table 1, Figure 1). The reductions were combined into specific heterozygous and homozygous models. In detail, these reactions refer to: a) intrinsic GTPase activity of Gα_GTP_, b) binding of Gα_GTP_ to PDE and resulting PDE activation, and c) shut-off of the photoresponse by RGS binding and RGS-catalyzed GTP hydrolysis. These reactions are highlighted in Figure 1 and listed in Table 1. In line with *in vitro* electrophysiological recordings on rods from transgenic mice, we monitored the following features of the photocurrent elicited by increasingly stronger flashes of light (Figure 3): a) rate of the activation phase, b) light sensitivity, and c) speed of the recovery phase. In detail, a) the rate of the activation phase was evaluated as the time needed to reach the maximum value of ΔJ after the delivery of the flash; b) light sensitivity was taken as the normalized response amplitude as a function of flash strength (Figure 3G and Figure 5B in Moussaif et al. [8]); and c) the speed of the recovery phase was evaluated as the time needed for the photocurrent to reestablish its dark value (ΔJ = 0) after a flash. In some cases, the parameters had to be eventually set equal to 0, while in other cases also more limited reductions led to modification of the output (Table 1). All the numerical simulations were carried out by means of Matlab, within the SBTOOLBOX2 framework [18] (http://www.sbtoolbox2.org/main.php) as already described [14].

### MD simulations: The structural models

The following PDB structures were selected as inputs of MD simulations: Gα_GTP_^WT^ (PDB code: 1TND [19], residue range 27–342), which is the GTP-bound form of Gα, and the Gα_GTP_-RGS-PDEγ^WT^ ternary complex (PDB code: 1FQJ [16]) involving Gα_GTP_ (amino acids from 28 to 344), RGS (i.e. the RGS domain of RGS9, amino acids 286 to 418) and the 42-amino acid C-terminal fragment of PDEγ (residues 46–87). Input structure setup required a number of modifications in the original crystal structures. As for 1TND, the original GTPγS analogue was replaced by GTP, as recently reported [20]. As for 1FQJ, the original Gα was indeed a chimera identical to Gα_t_ except for residues 216–294 which were replaced with the corresponding homologous region of Gα_i1_ (residues 220–298). The Gα_i1_ sequence was therefore mutated into the corresponding one in bovine Gα_t_. Moreover, the original GDP-AlF_4_^−^ was replaced by GTP.

All the simulated systems hold the Mg^2+^ ion together with the coordinating water molecules.

The native G protein in the Gα_GTP_^WT^ and Gα_GTP_-RGS-PDEγ^WT^ complexes was finally subjected to the substitution of aspartate for G38, in order to produce the pathogenic mutant (i.e. Gα_GTP_^G38D^ and Gα_GTP_-RGS-PDEγ^G38D^). *In silico* mutagenesis was performed by means of the Quanta software (www.accelrys.com).

### MD simulation: Set-up

MD simulations on the four systems, Gα_GTP_^WT^, Gα_GTP_-RGS-PDEγ^WT^, Gα_GTP_^G38D^ and Gα_GTP_-RGS-PDEγ^G38D^, were carried out by using the GROMACS4 simulation package [21] with the AMBER03 all atoms force field [22], [23]. The TIP3P water model was employed to describe the solvent. AMBER parameters to describe the GDP and GTP molecules were taken from the literature [24]. Depending on the dimensions of the systems, a variable number of Na^+^ and Cl^−^ ions were placed at optimum electrostatic positions in order to neutralize the system. In detail, the systems included: 63740 total atoms for Gα_GTP_^WT^ (19512 water molecules, 48 Na^+^ and 38 Cl^−^ ions); 63743 total atoms for Gα_GTP_^G38D^ (19511 water molecules, 49 Na^+^ and 38 Cl^−^ ions); 81906 total atoms for Gα_GTP_-RGS-PDEγ^WT^ (24609 water molecules, 60 Na^+^ and 50 Cl^−^ ions); 81882 total atoms for Gα_GTP_-RGS-PDEγ^G38D^ (24599 water molecules, 61 Na^+^ and 50 Cl^−^ ions).

Periodic Boundary Conditions (PBC) were applied by using an octahedric box as a unit cell, imposing a minimum distance of 12 Å between the solute and the box boundaries.

MD simulation setup is the same as the one recently employed to simulate a number of Ras GTPases [20]. All the input crystallographic structures were subjected to energy minimization keeping restricted the positions of the main chain atoms, the nucleotide, the Mg^2+^ cation and the coordinating water molecules. The systems were then equilibrated at 300 K for 4 ns of backbone restricted MD simulations. The Particle Mesh Ewald (PME) method was employed to compute the electrostatic interactions. Short range repulsive and attractive interactions were computed by using a Lennard-Jones potential with a cutoff of 10 Å. The LINCS algorithm [25] was used to constrain all bond lengths except those in water molecules, allowing for an integration time step of 2 fs through the leap-frog algorithm. The v-rescale thermostat [26] was employed to keep the system at a constant temperature of 300 K, by using a coupling constant (τ_t_) of 0.1 ps. The pressure of the system was kept fixed at 1 atm, using the Berendsen weak coupling algorithm [27] with a coupling constant (τ_p_) of 1 ps. The pre-equilibrated systems were then subjected to 100 ns of unrestrained isothermal-isobaric (T = 300K, P = 1 atm) MD simulations.

### MD analyses of the intrinsic flexibility

MD trajectories were subjected to a variety of analyses aimed at inferring a) the time series of a number of structural descriptors such as the SASA, b) the intrinsic flexibility of the systems (e.g. RMSD, RMSF, and Essential Dynamics (ED) or PCA), and c) potential correlations between structural descriptors and essential motions (i.e. FMA).

As for ED, resting on the assumption that the major collective modes of fluctuation dominate the functional dynamics of a system, information on such global motions can be inferred from the atomic fluctuations by means of the PCA. The latter allows the decomposition of the atomic fluctuations into a set of principal components (eigenvectors of the covariance matrix of positional fluctuations) that describe the concerted motions of these atoms (e.g. the Cα-atoms). The technique is based on the diagonalization of such covariance matrix producing a set of eigenvector and eigenvalue pairs in which the eigenvector and the eigenvalue describe, respectively, direction and amplitude of the concerted atomic motion (a mode). The atomic components of an eigenvector provide a quantitative measure of the participation of each Cα-atom to the collective motion described by the corresponding eigenvector. The subspace spanned by the major modes of collective fluctuations is accordingly often referred to as “essential subspace (ES)”. In the same framework, FMA is a technique to identify collective atomic motions related to a specific protein function. Given a large set of structures of one protein, for example from an MD trajectory, the method detects a mode that is maximally correlated to an arbitrary quantity of interest.

Except for FMA, which was carried out by using the GROMACS package, all these MD analyses were performed by means of the Wordom software [28]. As for PCA, the covariance matrices were built on the Cα-atoms of the isolated MD trajectories.

FMA [29] was carried out by using the Linear Mutual Information (LMI) estimator [30]. The structural descriptor correlated with the Principal Components (PCs) was the SASA calculated on selected Gα amino acids involved in Gα-PDEγ and Gα-RGS interactions. A number of PCs were probed.

Non bonded interaction energies for the nucleotide were monitored every 20 ps along the trajectory with GROMACS4.

### MD analyses of the structural communication

The structural communication (i.e. PSGs and shortest communication paths) in the four simulated systems was inferred by means of the graph-based approach proposed by Vishveshwara and coworkers [31] and defined as Protein Structure Network (PSN), that was recently implemented in the Wordom software [28]. With this approach, the dynamics of the system is taken into account in terms of occurrence of network components along the trajectory and of correlated motions [32]–[34].

A graph is defined by a set of points (nodes) and connections (edges) between them. In a PSG, each amino acid is represented as a node and these nodes are connected by edges based on the strength of non-covalent interactions between nodes [31]. The strength of interaction between residues i and j (I_ij_) is evaluated as a percentage given by the following equation:
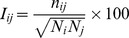
where I_ij_ is the percentage interaction between residues i and j; n_ij_ is the number of atom-atom pairs between the side chains of residues i and j within a distance cutoff (4.5 Å); N_i_ and N_j_ are normalization factors for residue types i and j, which take into account the differences in size of the side chains of the residue types and their propensity to make the maximum number of contacts with other amino acid residues in protein structures. The normalization factors for the 20 amino acids were taken from the work by Kannan and Vishveshwara [35]; the normalization values for GTP (derived from 3 heterotrimeric G proteins), Mg^2+^ (based on 4 heterotrimeric G proteins to properly describe the coordination of such ion in the system under study) and water (based on 5 structures, comprising G proteins and rhodopsin) were 361.3, 23.8 and 27.0, respectively. Thus, I_ij_ are calculated for all nodes, excluding i ± n, where n is a given neighbor cutoff of 3. An interaction strength cutoff I_min_ is then chosen and any residue pair ij for which I_ij_≥I_min_ is considered to be interacting and hence is connected in the PSG.

As previously demonstrated [31], the optimal I_min_ is the one at which the size of the largest cluster of nodes at I_min_ 0% halves. Incidentally a node cluster is a set of connected nodes in a graph. We approximated the I_min_ value to the second decimal place. The final I_min_ cutoffs were: 3.24% for Gα_GTP_^WT^, 3.23% for Gα_GTP_^G38D^, 3.52% for Gα_GTP_-RGS-PDEγ^WT^, and 3.58% for Gα_GTP_-RGS-PDEγ^G38D^. To build the PSG, only the edges present in at least 30% of the trajectory frames were used. Those nodes involved in at least four links are named as hubs.

Possible shortest communication paths or optimal paths (OPs) in the different Gα_GTP_ binary complexes as well as between wild type and mutated Gα_GTP_ and the other two proteins in the Gα_GTP_-RGS-PDEγ ternary complex were searched. All residue pairs except those at sequence distance ±5 were considered as path extremities (i.e. the first and last amino acids in the path). In detail, the number of intra-Gα amino acid pairs was 49770 for the wild type and mutated forms of isolated Gα_GTP_ and 50090 for the wild type and mutated forms of Gα_GTP_ in the ternary complex with RGS and PDEγ. Finally, 56350 amino acid pairs were considered to search all possible communication paths between wild type and mutated Gα_GTP_ and the two proteins in the ternary complex. Vishveshwara and co-workers implemented also the search for suboptimal paths (SOPs), alternate routes of communication, which can be computed by systematically removing all interactions of an OP node(s), thus forcing the traversal of a less than optimal path [36]. Since our path searches, different from those by Vishveshwara and co-workers [36]–[38], are not limited to a few selected node pairs but systematically consider almost all node pairs in a system, the additional search for SOPs would have been too costly in terms of computer time with the high risk to produce more noise than relevant information. For this reason our approach is dedicated exclusively to OPs.

The search for the shortest path(s) between pairs of nodes as implemented in the PSN-path module of Wordom relies on the Dijkstra's algorithm [39]. They were searched by combining PSN data with cross-correlation of atomic motions calculated by using the LMI method.

Following calculation of the PSG and of correlated motions (by means of the LMI method [30]), for each frame, the procedure to search for the shortest path(s) between each residue pair consists of a) searching for the shortest path(s) between each selected amino acid pair based upon the stable PSN connectivities, and b) selecting the shortest path(s) that contains at least one residue correlated (i.e., with a LMI cross-correlation ≥0.3) with either one of the two extremities. All the shortest paths that pass the filter of correlated motions are subjected to a further filter based upon path frequency, i.e. number of frames containing the selected path divided by the total number of frames in the trajectory. The relative number of amino acids holding correlated motions with either one of the two extremities is quantified by the correlation score, i.e. the ratio between the number of correlated amino acids and the path length; the latter excludes the two extremities.

Outcome of this stage is the total pool of paths for the system under study. Meta paths made of the most recurrent nodes and links in the path pool (i.e. global meta paths) are worth computing to infer a coarse/global picture of the structural communication in the considered system. In this study, meta paths were computed on the ensemble of paths with frequency ≥30%. For each link a recurrence score *r* is calculated using the following equation:
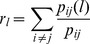
where *l* is a given link present in the considered set of shortest paths, *p_ij_* is the total number of shortest paths from node *i* to node *j* and *p_ij_(l)* is the total number of shortest paths from node *i* to node *j* that include link *l*. Finally, only those links with a recurrence score ≥30% of the highest score are used in the meta path representation.

## Supporting Information

Figure S1 **Details of GTP binding modes in Gα_GTP_ (top) and Gα_GTP_-RGS-PDEγ (bottom).** In both panels, the superimposed structures of wild type (violet) and mutated (green) forms are shown. The nucleotide is always colored by atom type. Only the amino acids that contribute the most to interactions with the nucleotide are shown in sticks. For those amino acids, which contribute through the backbone NH group, only the latter is shown. The mutated side chain is cyan. See the legend to Figure 2 for the labeling scheme.
(TIFF)

Figure S2 **Nucleotide-protein non bonded interaction energies averaged along the trajectories for Gα_GTP_ (A) and Gα_GTP_-RGS-PDEγ (B).** In both panels, violet bars refer to the wild type form and green bars to the mutated form. Vertical black bars indicate standard errors. Only the non bonded interactions whose average values along the simulations were smaller than −20 kJ mol^−1^ were plotted.
(TIFF)

Figure S3 **Cα-RMSD plots.** The time series of the Cα-RMSD with respect to the input structures concerning isolated Gα_GTP_ from 1TND, complexed Gα_GTP_ from 1FQJ, PDEγ from 1FQJ, and RGS from 1FQJ are shown. Violet refers to the wild type whereas green refers to the mutant.
(TIFF)

Figure S4 **Fragment analysis on the pool of paths generated by Gα_GTP_ (top) and Gα_GTP_-RGS-PDEγ (bottom) structures.** In both panels, violet bars refer to the wild type form and green bars to the mutated form. Fragment recurrence is the number of paths containing the given fragment divided by the total number of paths. On the abscissa, the nodes constituting the fragment are numbered according to the secondary structure nomenclature explained in the legend to Figure 2 and used throughout the text. Only fragments of length 3 were taken into account.
(TIFF)

Figure S5 **Global and coarse view of the communication pathways with high content of conserved amino acids.** The meta paths computed over those paths holding ≥50% of conserved amino acids are shown. They concern Gα_GTP_^WT^ and Gα_GTP_^G38D^ in their free state (A and B panels, respectively) as well as in ternary complex with both RGS and PDEγ (C and D panels, respectively), colored violet and green respectively. The width of each link is proportional to *r*, while the sphere diameter is proportional to the average *r* of the connecting link (see Methods for *r* definition). The α-helical and Ras-like domains are dark and light gray, respectively, the PDEγ binding site on Gα is aquamarine, RGS is orange and PDEγ is lemon-green. The mutation site is indicated by the red sphere.
(TIFF)

Figure S6 **Cα-RMSF profiles and Cα-atom projections.** **A.** The Cα-RMSF profiles from MD trajectories of Gα from Gα_GTP_-RGS-PDEγ^WT^ (violet) and Gα from Gα_GTP_-RGS-PDEγ^G38D^ (green) are shown. They refer to the 100000 frames constituting the 100 ns trajectory. The secondary structure elements are shown on the abscissa, following the Noel's nomenclature (see [19] in the text). **B, C, D, E.** The Cα-atom projections along the linear combination of the PCA-derived principal components, which describe the ES of the trajectories of Gα from Gα_GTP_-RGS-PDEγ^WT^ (B and C) and of Gα from Gα_GTP_-RGS-PDEγ^G38D^ (D and E) are shown. The ES is given by a variable number of eigenvectors that describe 90% of the total variance (sum of eigenvalues). The number of PCs used was 108 for B and C, and 103 for D and E. Cα-atom displacements are highlighted by color ranges from violet to blue or and from green to blue, respectively.
(TIFF)

Figure S7 **Cα-RMSF profiles.** The Cα-RMSF profiles from MD trajectories of PDEγ (top) and RGS (bottom) from Gα_GTP_-RGS-PDEγ^WT^ (violet) and Gα from Gα_GTP_-RGS-PDEγ^G38D^ (green) are shown. The secondary structure elements are shown on the abscissa.
(TIFF)
