# Supplementary figures and images for: Network and Atomistic Simulations Unveil the Structural Determinants of Mutations Linked to Retinal Diseases
Source: PLoS Comput Biol. 2013 Aug 29;9(8):e1003207. doi: 10.1371/journal.pcbi.1003207 (PMC3757061; doi:10.1371/journal.pcbi.1003207)

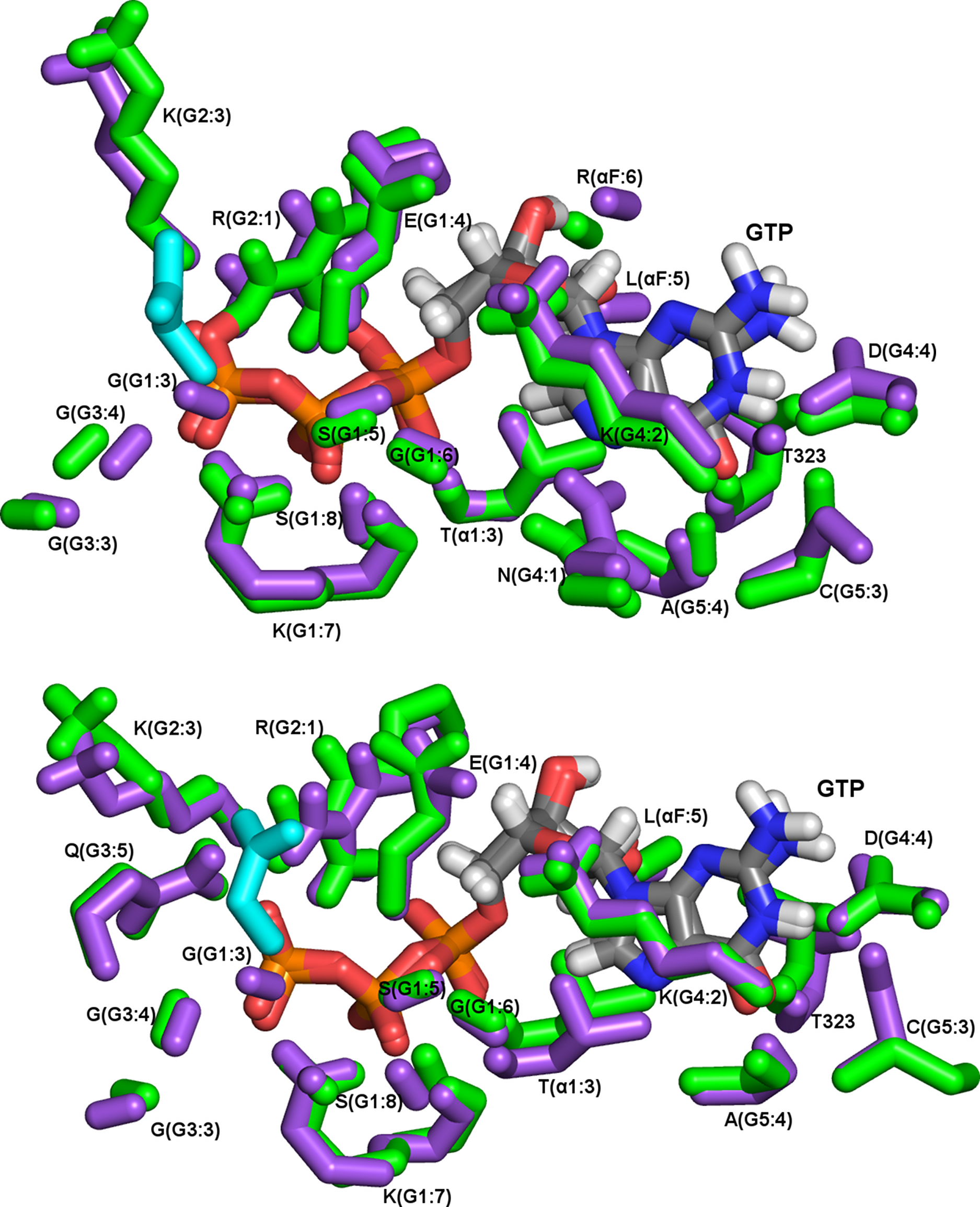

Supplement: Figure S1 — Details of GTP binding modes in GαGTP (top) and GαGTP-RGS-PDEγ (bottom). In both panels, the superimposed structures of wild type (violet) and mutated (green) forms are shown. The nucleotide is always colored by atom type. Only the amino acids that contribute the most to interactions with the nucleotide are shown in sticks. For those amino acids, which contribute through the backbone NH group, only the latter is shown. The mutated side chain is cyan. See the legend to Figure 2 for the labeling scheme. (TIFF) [file pcbi.1003207.s001.tiff]

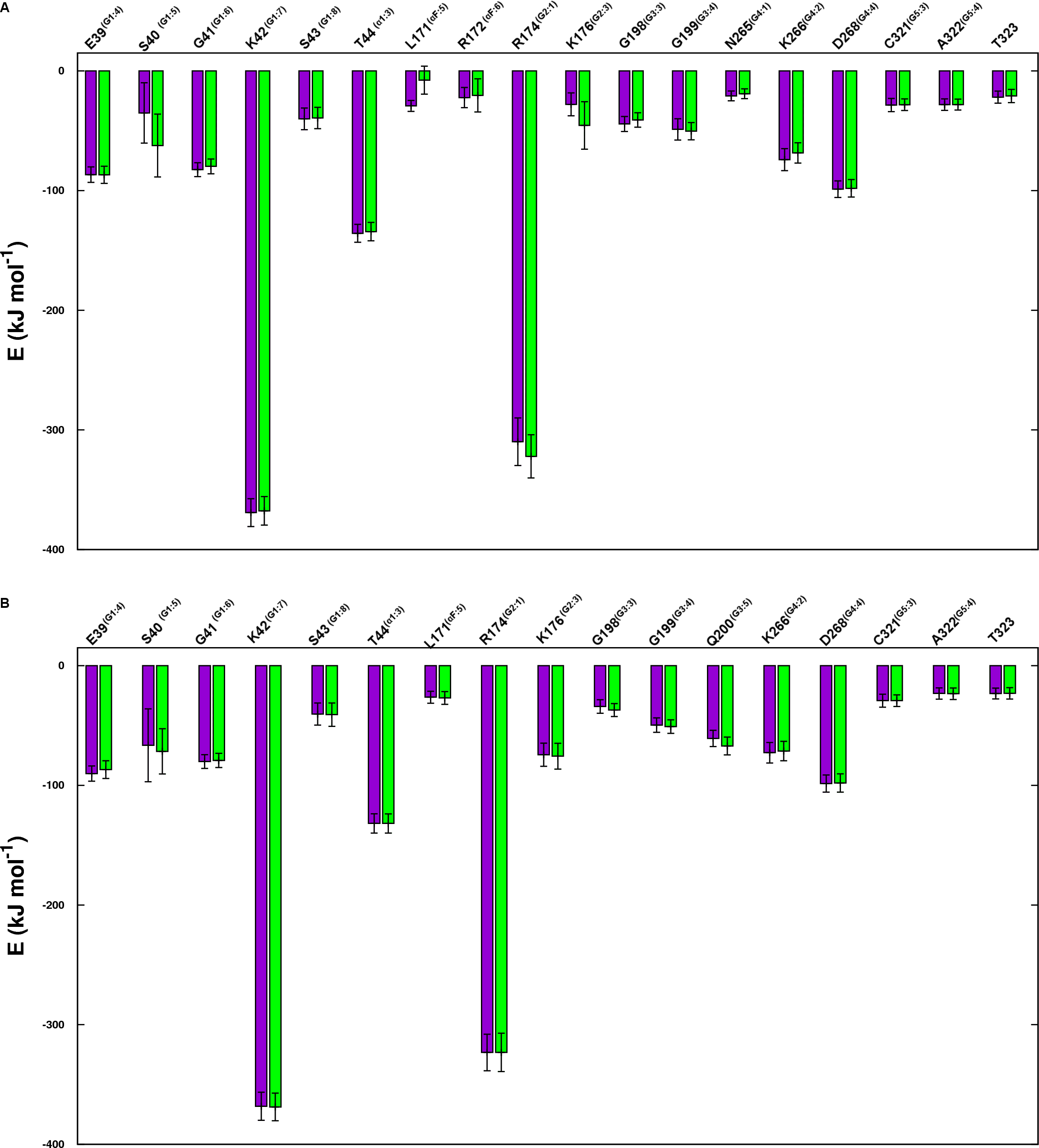

Supplement: Figure S2 — Nucleotide-protein non bonded interaction energies averaged along the trajectories for GαGTP (A) and GαGTP-RGS-PDEγ (B). In both panels, violet bars refer to the wild type form and green bars to the mutated form. Vertical black bars indicate standard errors. Only the non bonded interactions whose average values along the simulations were smaller than −20 kJ mol−1 were plotted. (TIFF) [file pcbi.1003207.s002.tiff]

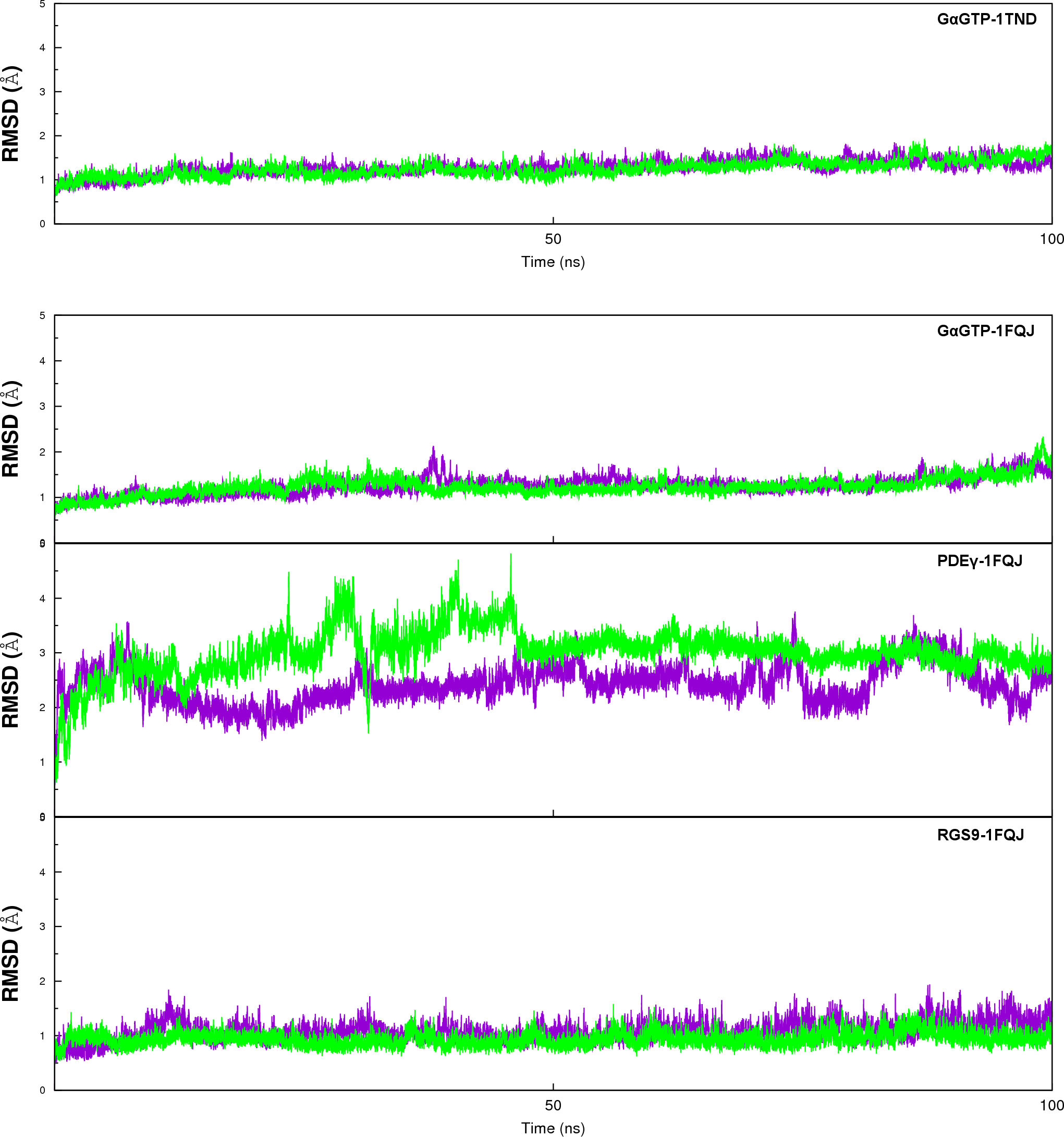

Supplement: Figure S3 — Cα-RMSD plots. The time series of the Cα-RMSD with respect to the input structures concerning isolated GαGTP from 1TND, complexed GαGTP from 1FQJ, PDEγ from 1FQJ, and RGS from 1FQJ are shown. Violet refers to the wild type whereas green refers to the mutant. (TIFF) [file pcbi.1003207.s003.tiff]

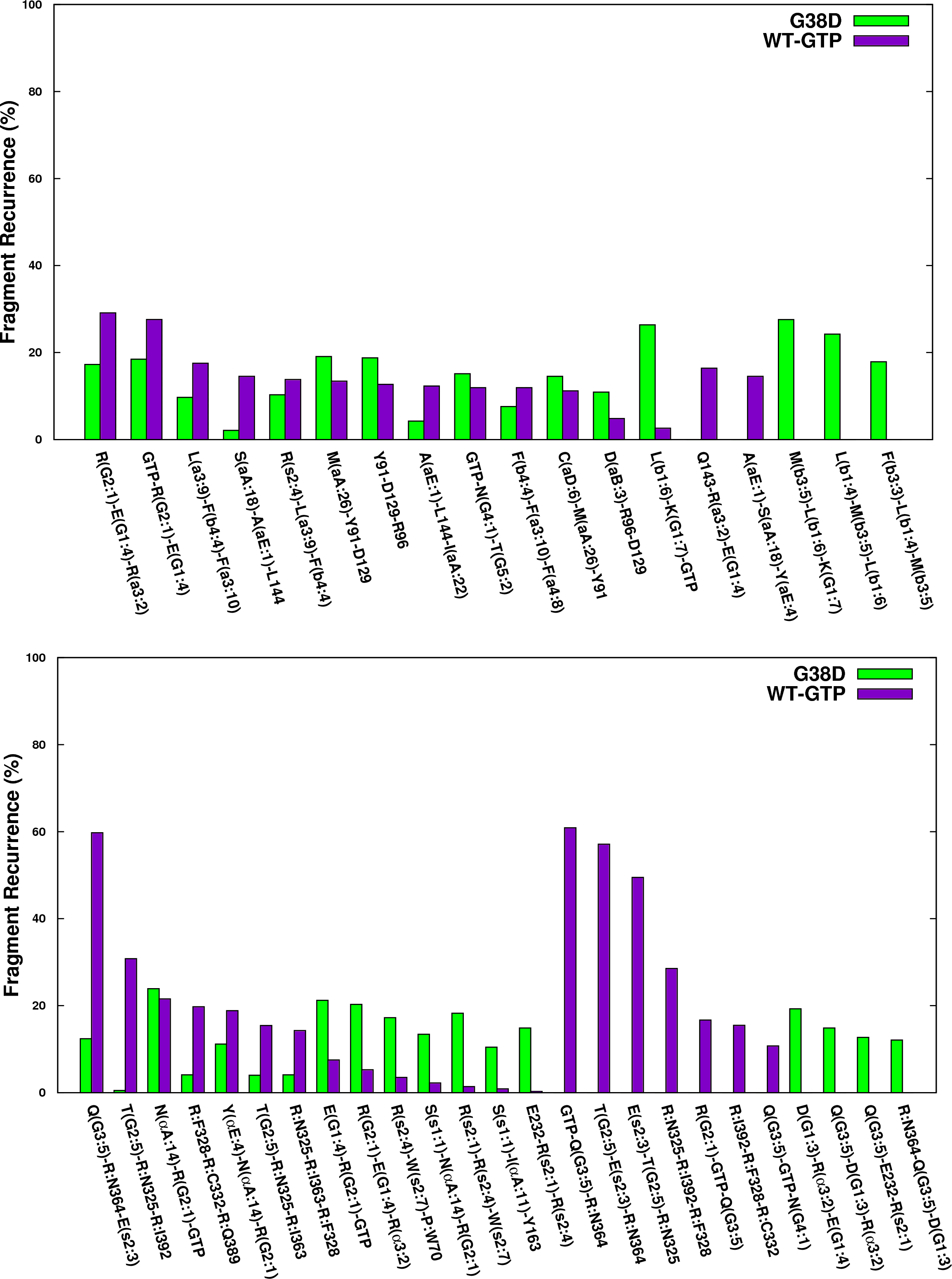

Supplement: Figure S4 — Fragment analysis on the pool of paths generated by GαGTP (top) and GαGTP-RGS-PDEγ (bottom) structures. In both panels, violet bars refer to the wild type form and green bars to the mutated form. Fragment recurrence is the number of paths containing the given fragment divided by the total number of paths. On the abscissa, the nodes constituting the fragment are numbered according to the secondary structure nomenclature explained in the legend to Figure 2 and used throughout the text. Only fragments of length 3 were taken into account. (TIFF) [file pcbi.1003207.s004.tiff]

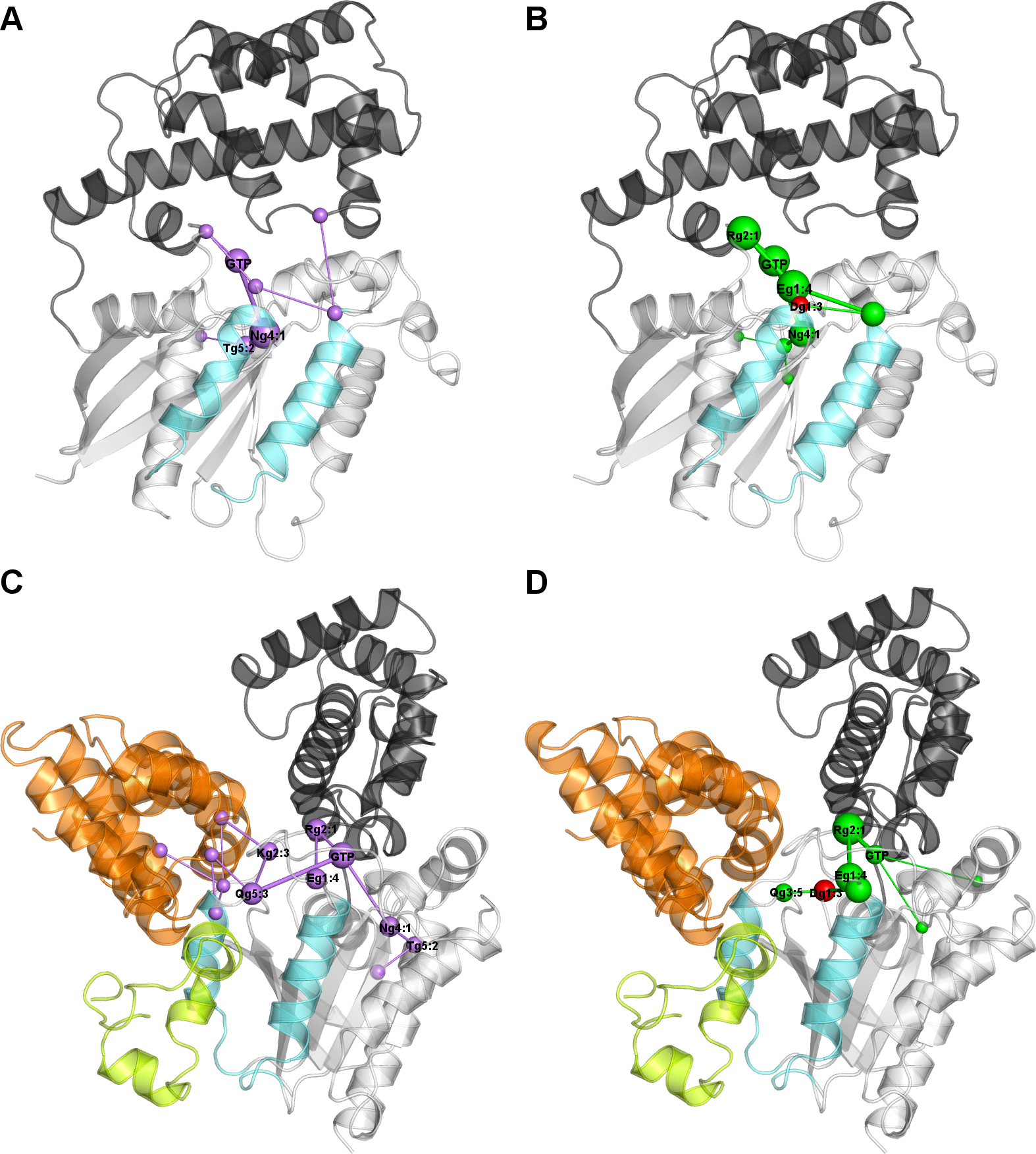

Supplement: Figure S5 — Global and coarse view of the communication pathways with high content of conserved amino acids. The meta paths computed over those paths holding ≥50% of conserved amino acids are shown. They concern GαGTP WT and GαGTP G38D in their free state (A and B panels, respectively) as well as in ternary complex with both RGS and PDEγ (C and D panels, respectively), colored violet and green respectively. The width of each link is proportional to r, while the sphere diameter is proportional to the average r of the connecting link (see Methods for r definition). The α-helical and Ras-like domains are dark and light gray, respectively, the PDEγ binding site on Gα is aquamarine, RGS is orange and PDEγ is lemon-green. The mutation site is indicated by the red sphere. (TIFF) [file pcbi.1003207.s005.tiff]

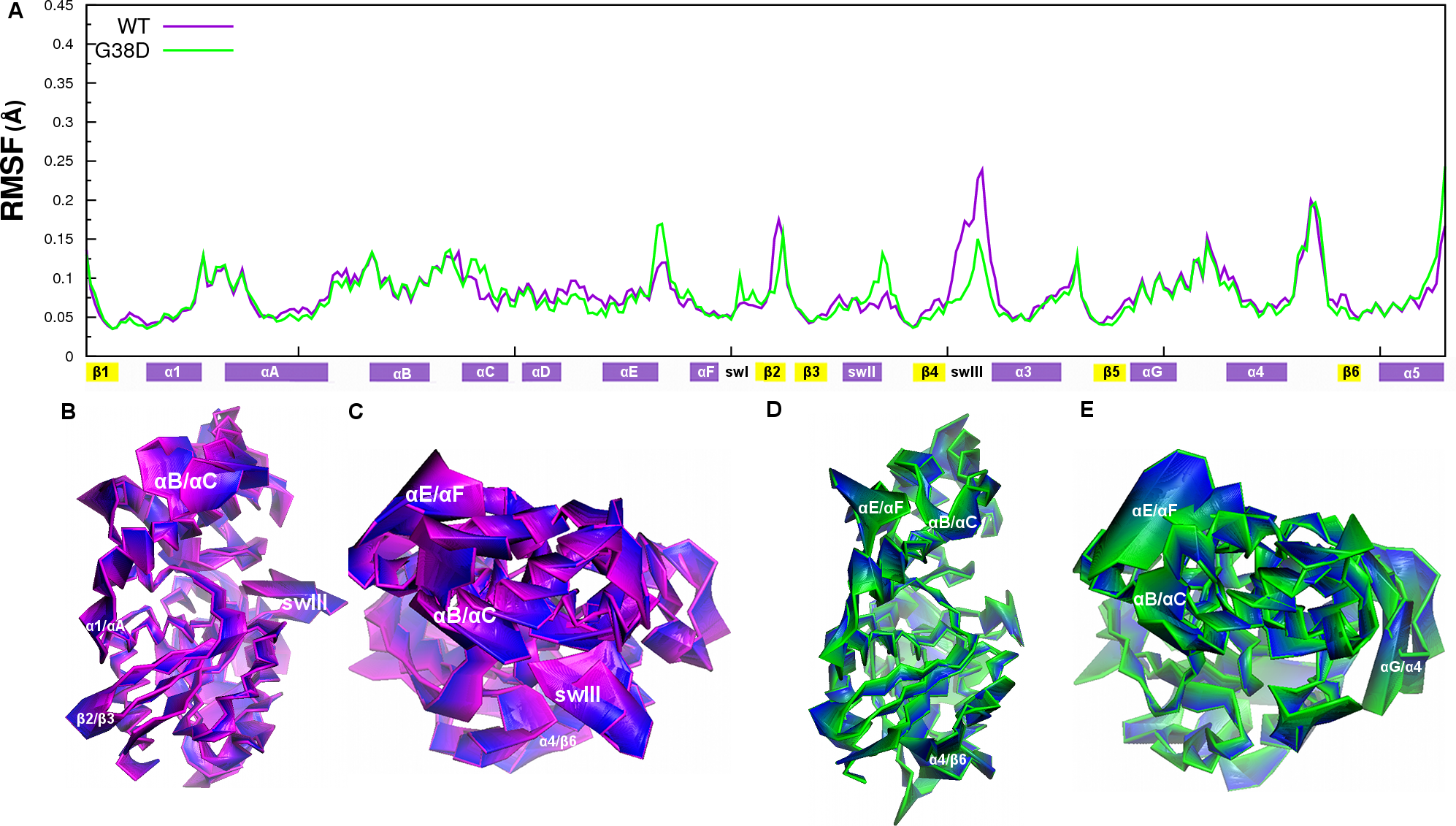

Supplement: Figure S6 — Cα-RMSF profiles and Cα-atom projections. A. The Cα-RMSF profiles from MD trajectories of Gα from GαGTP-RGS-PDEγWT (violet) and Gα from GαGTP-RGS-PDEγG38D (green) are shown. They refer to the 100000 frames constituting the 100 ns trajectory. The secondary structure elements are shown on the abscissa, following the Noel's nomenclature (see [19] in the text). B, C, D, E. The Cα-atom projections along the linear combination of the PCA-derived principal components, which describe the ES of the trajectories of Gα from GαGTP-RGS-PDEγWT (B and C) and of Gα from GαGTP-RGS-PDEγG38D (D and E) are shown. The ES is given by a variable number of eigenvectors that describe 90% of the total variance (sum of eigenvalues). The number of PCs used was 108 for B and C, and 103 for D and E. Cα-atom displacements are highlighted by color ranges from violet to blue or and from green to blue, respectively. (TIFF) [file pcbi.1003207.s006.tiff]

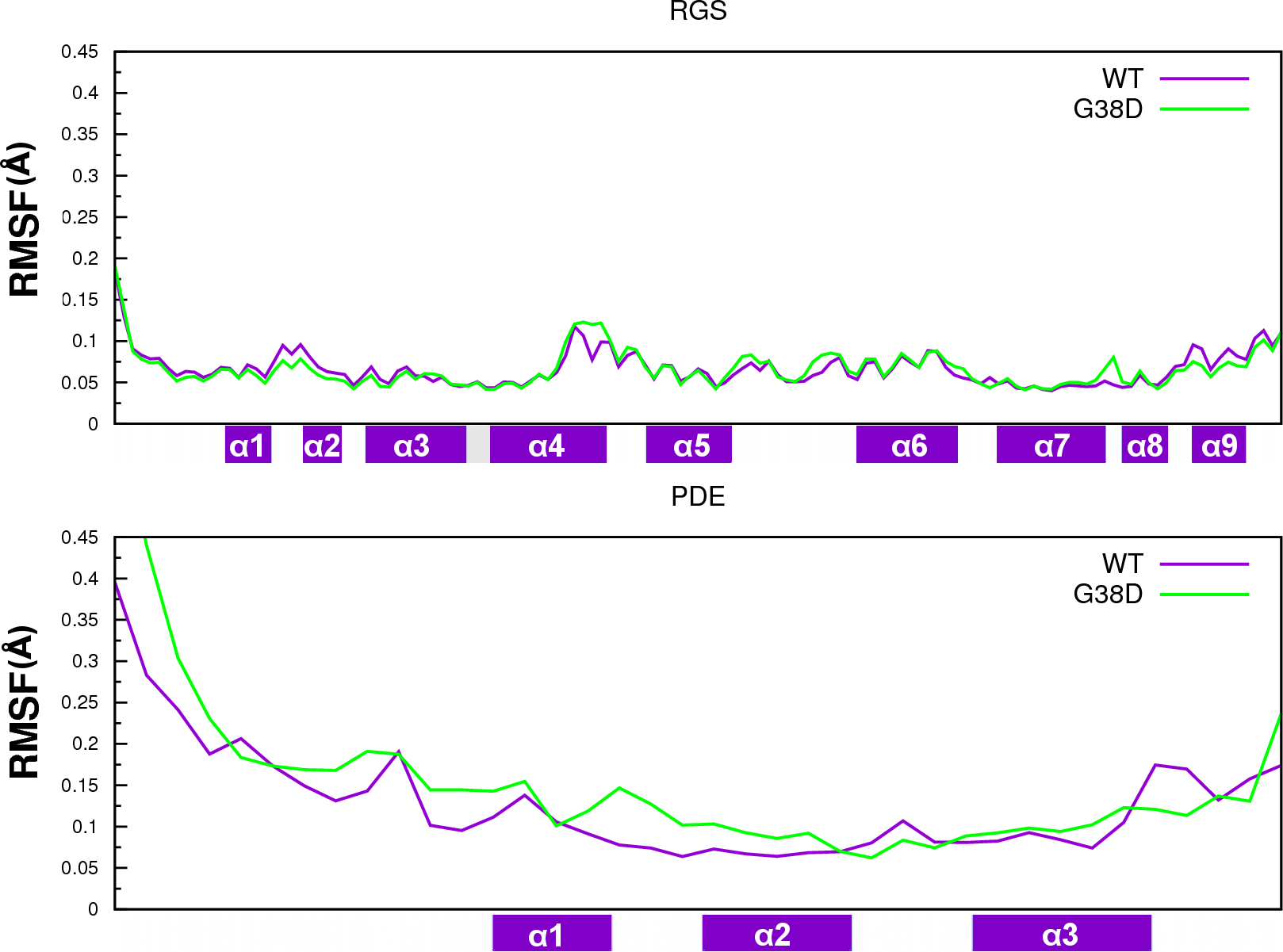

Supplement: Figure S7 — Cα-RMSF profiles. The Cα-RMSF profiles from MD trajectories of PDEγ (top) and RGS (bottom) from GαGTP-RGS-PDEγWT (violet) and Gα from GαGTP-RGS-PDEγG38D (green) are shown. The secondary structure elements are shown on the abscissa. (TIFF) [file pcbi.1003207.s007.tiff]
